# Trauma-related Therapeutic Procedures at Shohada Trauma Center in Tabriz

**DOI:** 10.5812/traumamon.7737

**Published:** 2013-01-15

**Authors:** Homayoun Sadeghi-Bazargani, Saber Azami-Aghdash, Behrad Ziapour, Reza Deljavan

**Affiliations:** 1 Neuroscience Research Center, Statistics and Epidemiology Department, Tabriz University of Medical Sciences, Tabriz, IR Iran; 2 Health Services Administration Department, Tabriz University of Medical Sciences, Tabriz, IR Iran; 3 Emergency Medicine Department, Ahwaz Jundishapur University of Medical Sciences, Ahwaz, IR Iran; 4 Injury Epidemiology and Prevention Research Center, Tabriz University of Medical Sciences, Tabriz, IR Iran

**Keywords:** Trauma, Wounds and Injuries, Accidents, Traffic

## Abstract

**Background:**

To decrease the burden of injuries it is essential to have an overview of trauma patterns and its management at regional trauma centers.

**Objectives:**

The aim of this study was to investigate some patterns of trauma and trauma-related therapeutic interventions at our trauma center.

**Materials and Methods:**

In a cross-sectional study, 19530 trauma cases admitted to the emergency department and hospital wards of Shohada University Hospital during 2007-2008 were assessed.

**Results:**

Of the 19530 trauma cases, 14960(76.7%) were males. Mean (SD) of age was 31(19.9) years. The elderly aged 65 and above, comprised 10% (1953) of the participants; while 44 were infants. Falls and traffic injuries were the most common cause of injuries among trauma patients. Most of the mortalities were men comprising 74% of the 57 deaths. Reduction of fractures and dislocations were the most common types of operations among trauma patients.

**Conclusions:**

Young men form the target group for possible interventions to decrease the burden of trauma following falls and traffic accidents.

## 1. Background

Trauma comprises a noticeable global burden of health problems affecting nearly every population in the world (1). It has been stated that 50% of people face traumatic events at least once throughout their lifetime (2). Lower and middle income countries (LMICs) suffer a high trauma morbidity and mortality; nevertheless, even in Europe and USA injuries are among the leading causes of death (3). Injuries, especially traffic injuries, are considered a major public health challenge (4). Every month about 2000 people die due to traffic accidents in Iran (5). Trauma is the second cause of mortality in Iran (6). To decrease the burden of injuries it is essential to have an overview of trauma patterns and management at regional trauma centers.

## 2. Objectives

The aim of this study was to assess patterns of trauma and trauma-related therapeutic interventions at the Shohada Trauma Center in the north-western Iran.

## 3. Materials and Methods

In a cross-sectional study, 19,530 trauma cases admitted to the emergency department and hospital wards of Shohada University hospital during 2007-2008 were assessed. Data were retrieved from the available electronic health information system. The system is run and regularly evaluated by expert staff. This health information system, registers minimal but essential information as follows:

Demographic data such as age and gender, Admission type, Referral history,Trauma coding based on international classification of diseases version 10 (ICD10). External causes of injuries coding based on ICD10, and Coding of procedures and operations based on ICD9-CM. 

The procedure codes that were separately analyzed in this study included; code 79 for fractures and dislocation operations; code 86 for dermal and subdermal operations and code 93 for application of orthopedic splints. The codes for external causes of injuries (first character code) that were separately analyzed based on ICD10, included:

“V (V01-V99) for transport accidents, W (W01-W99): W00-W19 for falls and W20-W99 for other external causes of accidental injury. X and Y:X00-X19 for various types of burn mechanisms, X20-X29 contact with venomous animals and plants, X30-X39 Exposure to forces of nature, X40-X49 Accidental poisoning by and exposure to noxious substances, X50-X57 Over-exertion, travel and privation, X58-X59 Accidental exposure to other and unspecified factors, X60- Y09 Intentional self-harm or assault, Y10-Y34 Event of undetermined intent, Y35-Y36 Legal intervention and operations of war, Y40-Y84 Complications of medical and surgical care, Y85-Y89 Sequelae of external causes of morbidity and mortality, Y90-Y98 Supplementary factors related to causes of morbidity and mortality classified elsewhere.” Data were entered into the computer and analyzed using Stata 11 statistical software package. Age was categorized through the analysis into five ages groups as; 0-6, 6-14, 14-30, 30-65, and above 65 years.

## 4. Results

**Figure 1.**
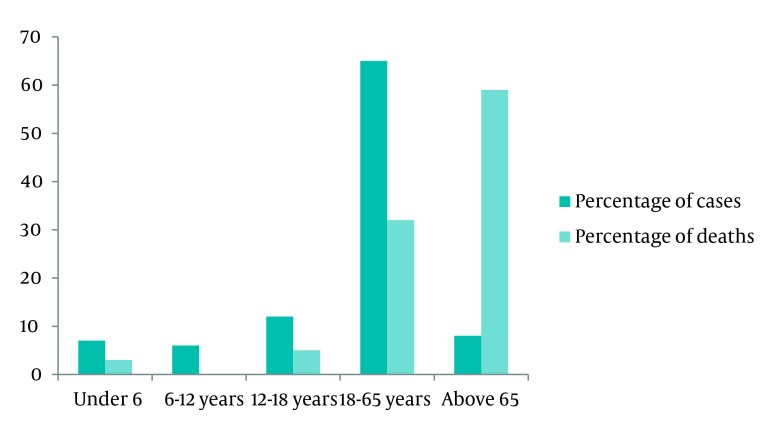
Relative Frequency of Trauma Deaths Due to Trauma in Various Age Groups

Of all the 19,530 trauma cases, 14960 (76.7%) were males. Mean (SD) of age was 31 (19.9) years. The elderly comprised about 10% of the participants and 44 were infants. Ninety-seven percent of the admissions were done through admission to EMS, the remainder being admitted directly to the hospital wards such as those referred from the clinics or private offices of the specialists who practice out of the hospital in parallel with their duties in the hospital. About 96% of patients were admitted for the first time. Two percent of victims were admitted to intensive care unit (ICU), while others were admitted in other wards of the hospital such as orthopedic surgery and neurosurgery wards. Fifty-seven fatal cases were registered out of the 19530 trauma cases. Most of the fatal cases belonged to the men comprising about 74% of the 57 deaths. Nevertheless, case fatality rates did not differ with gender. Figure 1 presents the distribution of relative frequency of trauma cases compared with the relative frequencies of death due to trauma in the various age groups. It indicates higher occurrence of mortality among the elderly.

**Figure 2.**
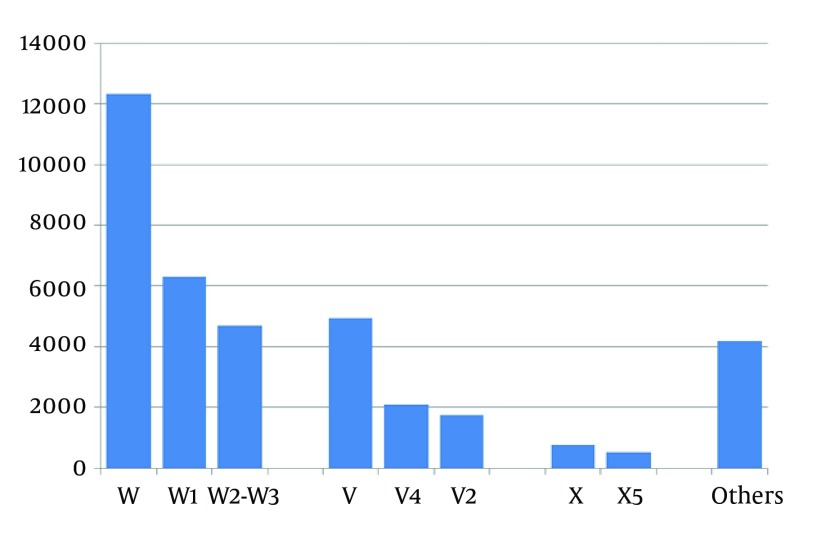
Distribution of the Most Common External Causes of Injuries Based on International Classification of Diseases Version 10 X-Axis: ICD 10 chapter 20 codes; Y-Axis: Number of trauma cases; V: Mostly intentional or iatrogenic harm; V2: Pedestrian injured by Bicycle; V4: Pedestrian injured by Heavy vehicles (such as buses); X: Exposure to burning agents, venomous animals and plants, poisoning, forces of nature and other unspecified factors; X5: Exposure to smoke, fire and flames; W: Other external causes of accidental injury including falls, drowning and exposure to mechanical forces and electrical current; W1: Same-level falling due to slipping tripping and stumbling; W2-3: Same-level falling on the ice, skiing and wrestling and other falls on the same level due to collision with, or pushing by, another person

Based on the international classification of diseases version 10, the frequency distribution of the common external causes of injuries are given in Figure 2. Moreover, distribution of the procedures used to treat the trauma patients is given in Table 1. Among the subgroups of code 79, the code item 79.12, belonging to the closed reduction of fracture with internal fixation, was the most common type involving 655 patients, followed by the code 79.02, belonging to closed reduction of fracture without internal fixation that involved 538 cases. Regardless of the main groups, application of splints was the most common type of operation followed by suturing of the skin and subcutaneous tissues of other sites.

**Table 1.** Therapeutic Interventions used to Manage Trauma Patients at the Shohada Trauma Center in North-Western Iran

Operation code	Definition	Frequency (%)
**79**	Reduction of fracture and dislocation	8594 (44.5)
**93**	Physical therapy, respiratory therapy, rehabilitation and related procedures	3555 (18.2)
**86**	Operation on skin and subcutaneous tissue	3090 (15.8)
**01-05**	Operations on the nervous system	3717 (18.97)
**Other codes**	Other bone operations, abdominal operations, vascular operations, Oral operations, Operations on muscle, tendon, and fascia, Catheter and tube drainage	574 (2.93)

## 5. Discussion

Reduction of fractures and dislocations was the most common type of operation among trauma patients in our study. This is because fractures and dislocations may occur both in traffic injuries and falls. Traffic injuries are the major types of injury mechanisms in Iran followed by burn injuries. However, this may not be the real pattern considering the fact that minor injuries are not usually captured in hospital based studies which form the majority of surveys in LMICs. For example minor burns comprise 95% of the burns and are usually overlooked in hospital-based studies (7).

 Among the middle-income countries, Iran has one of the highest mortality rates from traffic injuries (8). In Iran, there are about 70 deaths per day from road traffic injuries (9). Deaths from traffic injuries (30.0/100 000) in Iran have been reported by some researchers to be the highest in the world (4). Fall injuries and burn injuries are stated to be the two other major injuries of importance in Iran (4, 10, 11). However, in this study only cases of traffic injuries and falls were admitted. This is because burns are usually referred to an available separate specialty burn center in Tabriz. As with our study, previous research has also shown falls and traffic injuries to be the most common type of injuries among trauma patients (12-14).

Reduction of fractures and dislocations and application of splints were the most common operations and procedures in this study. This is quite consistent with the distribution of injuries based on the defined external causes. Also previous researches in Iran as well as American studies were in line with these findings (15, 16). The case-fatality reported in present study was lower compared to previous studies. Salami et al. in their study reported 208 mortalities out of 8000 hospital admissions (17). The fatality in that study was also lower than studies out of Iran. The lower case fatality may be due to the fact that both EMS patients as well as hospitalizations were enrolled, whereas, in previous studies only hospitalizations were studied (18, 19).

Falls due to slipping comprised the major cause of injury in our study while other Iranian studies showed different results (16, 20). This can be due to the fact that Shohada hospital is known for having famous orthopedic surgeons and orthopedic problems that are more common in falls were referred to this center. Most previous studies have shown the same-level falls to be more common than elevated falls (falls from height) in line with the results of our study (21, 22). Traffic injuries by heavy vehicles, bicycles and motorbikes were common types of injuries in this studies that were in line with other studies (20, 23). Most traffic accidents were pedestrian accidents that were consistent with previous study results (24, 25).

In present study, the injuries were four times more common among men. The male dominancy is a common finding in trauma studies indicating that, possibly due to their responsibilities, they are exposed to external causes of injuries more than women (24, 26, 27). Particularly in countries like Iran where women share a smaller role in transportation, this can normally be expected. This may not be true for other types of injuries such as burns and intoxications (7, 28-30).

### 5.1. Limitations

Like any other register-based study we were able to study only the few most important variables of interest due to the minimal principal data requirements in hospital registers. Under-registration of death counts is a rule in death registry systems and it is even higher in event-disease registers that also collect information about outcome measures including mortality.
